# Epidemiological and Clinical Behavior of Snakebite in the Pediatric Population, Associated with a Logistic Regression Model

**DOI:** 10.3390/clinpract15120228

**Published:** 2025-12-05

**Authors:** Cándida Revollo Vargas, Osnamir Bru-Cordero, Karina Pastor-Sierra

**Affiliations:** 1 Especialista en Pediatría, Facultad de Ciencias de la Salud, Universidad del Sinú E.B.Z., Montería 230001, Colombia; candidarevollo@unisinu.edu.co; 2 Dirección Académica, Universidad Nacional de Colombia, Kilómetro 9, Vía Valledupar-La Paz, La Paz 202010, Colombia; oebruc@unal.edu.co; 3 Grupo de Investigación Biomédicas y Biología Molecular, Universidad del Sinú E.B.Z., Montería 230001, Colombia

**Keywords:** snakebite, pediatric population, logistic regression

## Abstract

**Introduction:** Snakebite envenomation is recognized by the World Health Organization (WHO) as a neglected tropical disease. In Colombia, snakebites are frequent due to the diversity of ecosystems and snake species, and children represent a particularly vulnerable population. **Objective:** This study aimed to characterize the epidemiological and clinical behavior of snakebite envenomation in the pediatric population and to identify factors associated with its severity through the application of a multinomial logistic regression model. **Methods:** An exploratory analysis was conducted on 170 pediatric patients reported to the Public Health Surveillance System (SIVIGILA) and treated at San Jerónimo Hospital in Montería (HSJ). Sociodemographic and clinical data were collected, and a multinomial logistic regression model was applied to identify risk factors associated with the severity of envenomation. **Results:** Most cases occurred in children over 12 years of age (51.8%), and males were the most affected. The lower limbs were the most common site of the bite (87.6%). *Bothrops* was the main genus responsible. Non-medical practices, such as herbal poultices and potions, were reported in 28.2% of cases. Clinically, moderate envenomation was the most frequent (48.2%), with edema (88%) and pain (92%) as the main local manifestations, and nausea (36%) and vomiting (32%) as systemic manifestations. Cellulitis was the most common complication (24%). Student’s *t*-test showed a significant difference between complications and hospital stays lasting 3 to 7 days. The multinomial logistic regression explained 75% of the severity variability and showed that prior non-medical practices increased the risk of severe cases. **Conclusions:** Snakebite envenomation in children remains an important public health problem. The statistical model showed that non-medical practices are associated with a higher degree of severity.

## 1. Introduction

Snakebite envenomation refers to the injection of venom during a snakebite accident. It can cause local or systemic clinical manifestations or be asymptomatic. The latter occurs when the bite comes from a non-venomous snake or from a venomous snake without venom injection, known as a “dry bite” [1]. The World Health Organization (WHO) considers snakebite envenomation a neglected public health problem. On average, 5.4 million snakebites occur annually worldwide, with between 1.8 and 2.7 million cases of envenomation [2]. In addition, an estimated 81,410 to 137,880 deaths are reported annually, along with approximately three times as many amputations and other permanent disabilities [2,3].

Due to its geographical characteristics, Colombia ranks third in Latin America, after Mexico and Brazil, in the number of snakebite accidents [3,4]. In fact, in 2004, the Colombian National Public Health Surveillance System (SIVIGILA) classified snakebite envenomation as a notifiable event [5]. In 2018, the Colombian National Institute of Health (INS) reported a total incidence of 10.2 cases per 100,000 inhabitants, with 28.3% of cases occurring in individuals under 19 years of age. Of the 5434 cases recorded nationwide, the department of Córdoba accounted for 6.2%, ranking fifth in the country [6].

Globally, there are approximately 3000 snake species, of which only 270 are recorded in Colombia. Among them, only 18% are venomous [7], classified into two families: Viperidae (vipers) and Elapidae (elapids) [8]. In Colombia, most snakebite incidents are caused by the *Bothrops* genus, which belongs to the Viperidae family [6].

In terms of severity, snakebite envenomation is classified into categories based on the degree of envenomation and the snake genus, according to the surveillance protocol established by the National Institute of Health [9]. Clinical manifestations are similar in adults and children, but are more severe in the latter due to the higher amount of venom injected relative to body size [10]. Typical symptoms include inflammation and pain at the site of injury. Severe clinical signs such as hemorrhage, tissue necrosis, jaundice, seizures, coma, and/or paralysis may occur in a small percentage of cases. Complications such as cellulitis, pulmonary edema, compartment syndrome, acute renal failure, respiratory arrest, hypovolemic shock, anaphylaxis, and even death may also develop [9,10]. Snakebite disproportionately affects children in low-income settings and often results in permanent physical damage and psychological consequences [11]. Deaths can be related to several factors, but in pediatric patients, the main determinant is their smaller distribution volume, which allows for a rapid and severe dissemination of venom, leading to acute toxicity, coagulopathies, and severe local tissue damage [12].

In 2017, the departments of Córdoba, Antioquia, and Chocó reported a total of 34 deaths related to snakebite. In Córdoba, the municipalities with the highest frequency were Tierralta, Montería, and Puerto Libertador [13,14]. The *Bothrops* genus accounted for more than 60% of cases in Córdoba between 2007 and 2015, primarily affecting the lower limbs of individuals aged 10 to 19 years, highlighting the vulnerability of children and adolescents to snakebites and the risk of severe complications. Snakebite envenomation requires timely treatment, as survival rates are high when antivenom is administered promptly [15,16]. However, many cases experience delays in treatment, either due to long distances to health facilities or because patients undergo non-medical practices [14].

Despite the magnitude of the problem, research in Colombia has focused mainly on the general population and broader epidemiological aspects, with few studies dedicated to children and adolescents [7,8,9]. Consequently, there is a gap in the scientific literature that limits a comprehensive understanding of the clinical and epidemiological features of snakebite envenomation in pediatric populations.

The development of studies that integrate statistical models, such as multinomial logistic regression, is essential to identify factors associated with envenomation severity. This approach not only provides scientific evidence to guide clinical decision-making but also facilitates the design of prevention strategies, community education, and public health policies aimed at reducing morbidity, mortality, and long-term sequelae [7,8].

Accordingly, this study seeks to provide novel evidence on the epidemiological and clinical behavior of snakebite envenomation in the pediatric population of Córdoba, Colombia, addressing a critical knowledge gap and offering analytical tools. Furthermore, it is expected to serve as a foundation for future research exploring the long-term consequences of this condition.

## 2. Materials and Methods

### 2.1. Study Type

An exploratory data analysis study was conducted at the San Jerónimo Hospital in Montería (HSJ), a medium to high complexity healthcare center that serves as a reference for the department of Córdoba, Colombia, and its surrounding areas. The hospital is accredited to provide care for pediatric patients in the emergency department and hospitalization services.

The department of Córdoba, located in the northwestern region of Colombia (at approximately 8°45′ N, 75°53′ W), is characterized by a wide diversity of ecosystems, including both dry and humid tropical forests. These environmental conditions, combined with extensive agricultural and livestock areas, increase the risk of contact between rural populations and venomous snakes. According to reports from the National Institute of Health (INS) and the Public Health Surveillance System (SIVIGILA), between 2008 and 2016, the national incidence of snakebites ranged from 7.0 to 9.7 cases per 100,000 inhabitants, with a marked concentration in rural areas associated with agricultural activities [7,8,9]. During the same period, the Caribbean departments, including Córdoba, recorded rates higher than the national average. In Colombia, more than 90% of snakebite incidents are caused by snakes of the *Bothrops* genus, and in Córdoba, this figure exceeds 95%, with most cases linked to agricultural activities in remote rural areas [10,11]. Species of the *Viperidae* family produce venom characterized by the abundance of metalloproteinases, phospholipases A_2_ (PLA_2_), and serine proteinases, which constitute the main groups of toxins responsible for their biological effects [12].

### 2.2. Study Population

The total population consisted of 649 patients reported to SIVIGILA by HSJ with event code 100, corresponding to snakebite envenomation, in the period from 2016 to 2021. Out of these, 472 were over 18 years of age, and 177 were reported within the pediatric age range, which is the focus of this study. Subsequently, 7 patients were excluded due to data entry errors, resulting in a final sample of 170 patients. For this final sample, a review of electronic medical records was conducted using the Hospital Management Dynamics software to verify variables of interest for this study: sociodemographic (age, sex, department of origin, ethnic background), epidemiological (snake type), and clinical variables (bite location, non-medical practices, severity classification, local and systemic manifestations, local and systemic complications, delay in antivenom administration, and length of hospital stay).

### 2.3. Statistical Analysis

Data analysis was performed using R software, Version 4.2.0, Vigorous Calisthenics, Vienna, Austria (22). The results are expressed as mean and standard deviation. Furthermore, Student’s *t*-test or a *Kruskal–Wallis* test, as appropriate, was conducted to determine differences between the means of clinical variables, with a significance level of α = 0.05. The magnitude of risk was presented using odds ratios (OR) with their 95% confidence intervals. Additionally, a multinomial logistic regression model was fitted, where the response variable is the severity of the snakebite envenomation. It is worth noting that logistic regression models are statistical models used to understand the relationship between a qualitative dependent variable with more than two categories (multinomial logistic regression) and independent explanatory variables, which can be either qualitative or quantitative.

## 3. Results

The severity of the cases was classified following the surveillance protocol of the INS, 2018. According to this guideline, mild cases are those with limited local manifestations such as pain, edema, and erythema at the bite site without systemic involvement. Moderate cases present with more extensive local effects and may include mild systemic symptoms like nausea, vomiting, or dizziness. Severe cases are characterized by systemic complications, including hemorrhage, shock, acute renal failure, or neurological impairment. Using this classification allowed us to standardize the evaluation and ensure consistency when comparing clinical outcomes [5].

Table 1 shows the relationship between sociodemographic variables and the severity of the event. It is evident that 48.2% of patients who seek care at HSJ have a moderate snakebite envenomation. The age group most affected is those over 12 years old, accounting for 51.8% of cases. Males make up the majority of cases, with 69.4%. The department of Córdoba reports the majority of events, at 78.2%, followed by the department of Antioquia at 19.4. The majority of events occur in rural areas (84.7%), with most patients engaged in recreational activities at the time of the bite (46.4%), followed by hiking on trails (25.3%). The anatomical site most affected by snakebites is the lower limbs, accounting for 87.6% of cases. It is important to note that approximately 29% of patients are subjected to non-medical practices, especially those with moderate and severe bites. These practices include the use of tight tourniquets or ligatures, skin incisions with the intent of extracting the venom, direct suction of the wound, the application of substances such as gasoline or kerosene, the use of plant-based poultices, the application of ice or fire, and even the ingestion of alcoholic beverages or remedies prepared by traditional healers [17,18,19]. In over 70% of cases, information about the offending snake species was available, with the *Bothrops* genus being the most common, and 5.3% of accidents were attributed to the Crotalus genus. In 26.5% of cases, the genus could not be identified.

Table 2 presents the distribution of the clinical variables recorded. Edema and pain were the predominant local manifestations across all severity levels, occurring in 88% (150 cases) and 92% (157 cases) of patients, respectively. Among systemic manifestations, nausea was observed in 61 patients (36%) and vomiting in 54 (32%), both present across all severity categories. In moderate and severe cases, the most relevant systemic signs were gingival bleeding, reported in 24 moderate cases (60%) and 15 severe cases (38%); hematemesis, in 8 moderate (44%) and 10 severe cases (56%); and oliguria, in 6 moderate (33%) and 11 severe cases (61%). Cellulitis was the most frequent local complication, identified in 16 moderate (39%) and 22 severe cases (54%). The most common systemic complications were acute anemia (9 cases) and compartment syndrome (8 cases). Additionally, septic shock developed in six patients.

Table 3 presents the variables related to antivenom administration, surgical treatment, and hospital stay. Antivenom was administered to 90% of patients, except for those classified as non-envenomation, as well as two moderate cases and five mild cases. The most common time interval between the snakebite and antivenom administration was 2 to 6 h (47%), although 37 patients (23%) received treatment more than 12 h after the bite. Based on Table 3, the total number of cases that required surgical intervention was 13, of which 11 were classified as severe and 2 as moderate cases. Thirteen patients (8%) experienced adverse reactions following antivenom administration.

Regarding hospital stay, most patients with moderate envenomation remained hospitalized for 3 to 7 days, whereas in severe cases, the majority stayed for 8 days or longer. In contrast, most mild cases required hospitalization for 2 days or less.

Student’s *t*-test indicated a significant difference between the clinical variable “complications,” the time of antivenom application (more than 12 h), and a hospital stay between 3 and 7 days (Table 4). On the other hand, the variable “surgical treatment” showed a significant difference in cases with a hospital stay longer than 8 days.

Table 5 presents the discrete response model, specifically a multinomial logistic regression model, where the response variable is the severity level of snakebite envenomation (severe, moderate, mild, and non-envenomation), with non-envenomation as the reference category. It models the transition from one category to another by measuring the effects of the study’s covariates: age, gender, days of hospital stay, and non-medical practices.

For the estimations in this model, all the model assumptions were previously tested, and it was found that the model captures 75% of the variability in the response to be modeled. All tests turn out to be significant for a *p*-value less than 0.05.

The interpretation of odds ratios (ORs) provides insight into the strength and direction of the associations between explanatory variables and the severity of snakebite envenomation. For instance, the model showed that patients who underwent non-medical practices prior to hospital admission had an OR of 0.15 when comparing severe envenomation to non-envenomation. This means that the probability of a case being classified as severe was approximately 6.6 times higher (1/0.15) among individuals exposed to such practices, highlighting their negative impact on clinical outcomes

## 4. Discussion

Snakebites in the pediatric population are a public health event that requires increased attention from health authorities in Colombia. Its importance has been underestimated, and despite the creation of notification protocols and the evident exponential growth of cases over time, there is likely underreporting of this event. The variety of ecosystems and various environmental conditions, such as humidity and temperature, make Colombia the third richest country in snake species, exposing its inhabitants to a higher incidence of snakebites [20,21].

The HSJ in Montería, a center of medium and high complexity, reported a total of 649 snakebite cases to SIVIGILA during the study period, of which 27% corresponded to the pediatric age group, consistent with the findings of Lainez-Mejia et al. [22] and García-Willis et al. [23], where an average of 28% of cases involved individuals under 18 years old. This demonstrates that snakebites are a common occurrence in the pediatric population. These events occur in all pediatric age groups, with a lower incidence in children under 5 years, possibly because they spend more time at home and under the supervision of their caregivers. In this study, the highest number of cases occurred in children over 12 years old, which could be explained by their increased likelihood of playing outdoors, encountering snakes out of curiosity, and engaging in fieldwork.

Similar to other publications [16,19,24], males were more affected, the lower extremities were the most commonly involved, and the most common place of occurrence was the rural area. This can be attributed to the fact that the most common snake species in the study area inhabit humid forests, cultivated areas, and savannas. They can also be found among leaf litter, tree roots, or burrows [7]. This is why when children accompany their parents for agricultural and livestock activities, they are exposed to such accidents. In addition, the lack of protective gear, like boots, is very common, which could reduce the incidence. Furthermore, children often walk on trails to get to school or visit close relatives.

Table 1 presents the distribution, indicating that 28.2% of the children underwent prior non-medical practices, which is comparable to national reports [12] and in line with what was published by Márquez et al. [25] in a study on the population of Sucre, where 34.7% of their patients received these native treatments. These types of practices are carried out by healers or herbalists. Among the most common in this study are the use of potions, herbal poultices, the application of suction, incisions, and oral suction. These practices have their roots in Native American traditions but are also due to a lack of medical resources in many instances and, in other cases, to a lack of education about snakebite. Their use can delay medical attention and likely increases the risk of morbidity and mortality [15,18,26]. Although seasonality was not directly evaluated in our study results or tables, recent research indicates that most pediatric snakebite cases tend to cluster during the rainy seasons, particularly in May–July and November–December. This aligns with evidence highlighting the influence of rainfall and climatic factors on snakebite risk [20,21].

The Viperidae family is responsible for the majority of snakebite incidents, especially the *Bothrops* genus. This can be explained by the fact that multiple species of this genus are distributed throughout the country, consistent with reports from the INS [2,27] and other publications [17,19,20,25].

Additionally, these snakes are known to be aggressive and will not hesitate to attack, even if not provoked, solely out of feeling threatened [28]. Recent evidence supports these findings, as highlighted by Marriaga [13], where it is noted that the mentioned genus is the most common, accounting for 62.8% of cases in the department of Córdoba.

Most of the accidents were classified as moderate, which aligns with the majority of internationally published studies [9,15,29] and national studies [18]. As for local manifestations, most of the children experienced pain and swelling, consistent with findings from several authors [11,17,19,25,30]. However, these manifestations depend on the severity of the envenomation. For instance, a significant percentage of patients exhibited nausea and vomiting as their primary systemic symptoms, similar to the findings described by other authors [16,17,19,31].

It is important to highlight that gingival bleeding was the primary hemorrhagic manifestation in this study, which is consistent with what Otero et al. reported [17]. It is noteworthy that, despite all patients classified as moderate and severe envenomations receiving prophylactic antibiotic treatment as per protocol, cellulitis and abscesses were the main local complications found in these groups, with the latter being more frequent in patients classified as severe. These complications align with various studies [10,24,25,28,32,33]; 7.6% (Table 3) of the patients required surgical treatment, with the most common procedures being debridement, fasciotomy, and grafts. These results are consistent with previous research conducted in Colombia and other endemic countries, where severe local complications—such as tissue necrosis and compartment syndrome—are frequently associated with the need for surgical interventions [17,32,33].

The early administration of polyvalent antivenom is crucial for successful treatment when the appropriate dose is used based on the envenomation severity and considering the manufacturer’s guidelines. It is important to clarify that antivenom neutralizes circulating venom but does not modify established lesions resulting from its action [8]. Table 3 indicates that, in this study, 47% of patients received polyvalent antivenom within the first 2 to 6 h, but 42% experienced a delay of more than 7 h for its application, which differs from what Feitosa et al. [34] and Mendez et al. [10] reported, where, on average, 70% received antivenom before 6 h. This could be explained by the fact that many of these patients are located in dispersed rural areas, limiting their access to first-level healthcare facilities, where the appropriate treatment might not be available, and they may need to be referred to more complex centers such as HSJ. Additionally, many others are subjected to non-medical practices beforehand. Furthermore, potential differences in antivenom availability between Colombia, Brazil, and Honduras should be considered. Eight percent (8%) of the patients experienced an adverse reaction to antivenom application, consistent with national reports from the INS [12]. Most of these reactions were local, and there was no associated mortality. It is worth mentioning that these reactions were managed with epinephrine, antihistamines, and corticosteroids, and antivenom administration was subsequently continued without further complications. At this point, it is essential to note that none of the data sources consulted (SIVIGILA and clinical records) provided complete information regarding the manufacturer and the number of vials used in each envenomation case.

The multinomial logistic regression model (Table 5) showed that the severity of snakebite envenomation was significantly associated with the length of hospital stay, patient sex, and prior exposure to non-medical practices. Each additional day of hospitalization increased the likelihood of presenting severe envenomation by 15.7 times compared to non-envenomation cases, consistent with clinical studies showing that greater severity implies longer hospital stays in children admitted for snakebite envenomation [35,36]. Likewise, boys had a 1.59 times higher risk of developing severe envenomation compared to girls, which is consistent with recent observations attributing this difference to increased outdoor activities among male children [36,37]. Prior exposure to non-medical practices showed an inverse association with severity (OR = 0.15), in contrast to findings from contexts such as Sri Lanka, where such practices are usually linked to poorer clinical outcomes [37,38]. This discrepancy highlights the need to further investigate the sociocultural and contextual determinants of pre-hospital management, particularly in rural and remote communities.

## 5. Conclusions

The incidence of snakebite envenomation in the pediatric population has shown a significant increase over recent years, accounting for approximately 30% of all cases. This research revealed that the lower extremities are the most commonly affected body part, males have a higher incidence, and residents of rural areas are more affected. This suggests that children living in rural areas are at a higher risk of snakebites, especially during recreational activities or even while walking on trails. The primary offending snake genus is *Bothrops*; however, in 26.5% of cases, the responsible snake could not be identified, which could impact decision-making regarding the use of antivenom. Edema and pain are the most frequent local manifestations across all severity levels, while gingival bleeding is the primary hemorrhagic manifestation. Skin and soft tissue infections are the most common local complications despite prophylactic antibiotic therapy for moderate and severe cases. Early administration of antivenom reduces hospital stays, and the proposed model demonstrated that non-medical practices are associated with a higher risk of severity.

Limitations: The study was conducted in a referral hospital in the department of Córdoba; therefore, the findings may not be fully generalizable to other regions of Colombia, and underreporting of mild cases that did not reach healthcare services is possible. In addition, the COVID-19 pandemic may have influenced healthcare access and reporting during the study period.

## Figures and Tables

**Table 1:** Sociodemographic characteristics of the pediatric population with snakebite envenomation by severity level.

	Severity Frequency (%)				
	Severe	Moderate	Mild	Non-Envenomation	Total
Patient Number					
	36 (21.2)	82 (48.2)	40 (23.5)	12 (7.1)	170 (100)
Age Range					
<2	1 (16.7)	2 (33.3)	2 (33.3)	1 (16.7)	6 (3.5)
>3 or <5	4 (20)	10 (50)	5 (25)	1 (5)	20 (11.8)
>6 or <11	12 (21.4)	28 (50)	13 (23.2)	3 (5.4)	56 (32.9)
≥12	19 (21.6)	42 (47.7)	20 (22.7)	7 (8)	88 (51.8)
Gender					
Male	30 (25.4)	52 (44.1)	28 (23.7)	8 (6.8)	118 (69.4)
Female	6 (11.5)	30 (57.7)	12 (23.1)	4 (7.7)	52 (30.6)
Department of occurrence					
Antioquia	12 (36.4)	16 (48.5)	4 (12.1)	1 (3)	33 (19.4)
Bolívar	1 (100)	-	-	-	1 (0.6)
Choco	1 (50)	1 (50)	-	-	2 (1.2)
Córdoba	22 (16.5)	64 (48.1)	36 (27.1)	11 (8.3)	133 (78.2)
Sucre	-	1 (100)	-	-	1 (0.6)
Area of occurrence					
Urban	2 (7.7)	8 (30.8)	12 (46.2)	4 (15.4)	26 (15.3)
Rural	34 (23.6)	74 (51.4)	28 (19.4)	8 (5.6)	144 (84.7)
Ethnicity					
Indigenous	2 (22.2)	5 (55.6)	2 (22.2)	-	9 (5.3)
Afro-Colombian	-	1 (100)	-	-	1 (0.6)
Othres	34 (21.3)	76 (47.5)	38 (23.8)	12 (7.5)	160 (94.1)
Activity-Bite					
Water-related activity	1 (25)	1 (25)	-	2 (50)	4 (2.4)
Agricultural activity	4 (30.8)	5 (38.5)	4 (30.8)	-	13 (7.6)
Hijing on trails	13 (30.2)	21 (48.8)	9 (20.9)	-	43 (25.3)
Domestic chores	2 (8.7)	14 (60.9)	4 (17.4)	3 (13)	23 (13.5)
Recreation	15 (19)	36 (45.6)	22 (27.8)	6 (7.6)	79 (46.5)
Others	1 (12.5)	5 (62.5)	1 (12.5)	1 (12.5)	8 (4.7)
Bite site					
Head	1 (14.3)	3 (42.9)	2 (28.6)	1 (14.3)	7 (4.1)
Fingers (Hand)	-	3 (75)	1 (25)	-	4 (2.4)
Toes (Foot)	-	-	1 (100)	-	1 (0.6)
Back	-	-	-	1 (100)	1 (0.6)
Buttocks	-	-	1 (100)	-	1 (0.6)
Lower limbs	33 (22.1)	74 (49.7)	33 (21.1)	9 (6)	149 (87.6)
Upper limbs	2 (28.6)	2 (28.6)	2 (28.6)	1 (14.3)	7 (4.1)
Non-Medical Practices					
No	19 (15.6)	58 (47.5)	35 (28.7)	10 (8.2)	122 (71.8)
Yes	17 (35.4)	24 (50)	5 (10.4)	2 (4.2)	48 (28.2)
Snake Species/Genus					
*Bothrópico*	28 (24.1)	57 (49.1)	26 (22.4)	5 (4.3)	116 (68.2)
*Crotalico*	2 (22.2)	7 (77.8)	-	-	9 (5.3)
Unidentified	6 (13.3)	18 (40)	14 (31.1)	7 (15.6)	45 (26.5)

**Table 2:** Clinical characteristics of snakebite envenomation by severity level, recorded in the study population.

	Severity Frequency (%)				
	Severe	Moderate	Mild	Non-Envenomation	Total
Local Manifestations					
Edema	36 (24)	82 (55)	27 (18)	5 (3)	150 (88)
Pain	36 (23)	81 (52)	32 (20)	8 (5)	157 (92)
Erythema	21 (36)	32 (54)	6 (10)	-	59 (35)
Blisters	16 (64)	7 (28)	1 (4)	1 (4)	25 (15)
Bruising	23 (40)	26 (46)	7 (12)	1 (2)	57 (34)
Bruises	4 (100)	-	-	-	4 (2)
Systemic Manifestations					
Nausea	25 (41)	32 (52)	3 (5)	1 (2)	61 (36)
Vomiting	20 (37)	31 (57)	3 (6)	-	54 (32)
Gingival bleeding	15 (38)	24 (60)	1 (3)	-	40 (24)
Nosebleed	3 (60)	1 (20)	1 (20)	-	5 (3)
Hematemesis	10 (56)	8 (44)	-	-	18 (11)
Oliguria	11 (61)	6 (33)	1 (6)	-	18 (11)
Hematuria	8 (73)	2 (18)	1 (9)	-	11 (6)
Complications					
Cellulitis	22 (54)	16 (39)	3 (7)	-	41 (24)
Abscess	10 (67)	5 (33)	-	-	15 (9)
Necrosis	8 (73)	3 (27)	-	-	11 (6)
Compartment syndrome	8 (100)	-	-	-	8 (5)
Acute anemia	9 (100)	-	-	-	9 (5)
AKI	5 (100)	-	-	-	5 (3)
Séptic shock	6 (100)	-	-	-	6 (4)

AKI: acute kidney injury.

**Table 3:** Variables related to the administration of antivenom, surgical treatment, and hospital stay.

	Severity Frequency (%)				
	Severe	Moderate	Mild	Non-Envenomation	Total
Antivenom Administration					
Yes	36 (24)	80 (52)	35 (23)	2 (1)	153 (90)
No	-	2 (12)	5 (29)	10 (59)	17 (10)
Time Between Bite and Antivenom Administration					
≤1	2 (9)	13 (59)	7 (32)	-	-
Between 2 and 6	15 (20)	45 (60)	14 (19)	1 (1)	75 (47)
Between 7 and 12	9 (29)	12 (39)	9 (29)	1 (3)	31 (19)
≥12	14 (19)	13 (17)	9 (12)	1 (1)	37 (23)
Not applicable	-	2 (11)	5 (26)	10 (63)	17 (11)
Adverse Reactions to Antivenom					
Yes	4 (31)	4 (31)	4 (31)	1 (8)	13 (8)
No	32 (23)	76 (54)	31 (22)	1 (1)	140 (82)
Not applicable	-	2 (12)	5 (29)	10 (59)	17 (10)
Surgical Treatment					
Amputation	2 (100)	-	-	-	2 (1)
Debridement	3 (60)	2 (40)	-	-	5 (3)
Fasciotomy	1 (100)	-	-	-	1 (<1)
Fasciotomy and Graft	1 (100)	-	-	-	1 (<1)
Debridement, Fasciotomy, and Graft	4 (100)	-	-	-	4 (2)
Days of Hospital Stay					
≤2	4 (8)	9 (19)	24 (50)	11 (23)	48 (28)
Between 3 and 7	13 (14)	64 (68)	16 (17)	1 (1)	94 (55)
≥8	19 (68)	9 (32)	-	-	28 (16)

**Table 4:** Association of complications and treatment with sociodemographic variables, antivenom administration, and hospital stay.

	Complications		Mean ± SD	OR	*p*-Value	Surgical Treatment		Mean ± SD	OR	*p*-Value
	YES *n* (%)	NO *n* (%)				YES *n* (%)	NO *n* (%)			
Age (years)										
1–2	2 (16.7)	10 (83.3)	1.5 ± 0.5	0.04	0.138	-	6 (100)	1.5 ± 0.5	0.44	1
3–5	11 (27.5)	29 (72.5)	4.3 ± 0.7	0.13	0.075	2 (10)	18 (90)	4.3 ± 0.7	0.14	0.29
6–11	26 (23)	86 (77)	9.1 ± 1.6	0.49	0.849	8 (14)	48 (86)	9.1 ± 1.6	0.54	0.914
>12	38 (22)	137 (78)	14.6 ± 1.7	1.1	0.29	3 (3.4)	85 (96.6)	14.6 ± 16	0.77	0.778
Gender										
Male	56	180	-	2.27	-	2 (3.8)	50 (96.2)	-	0.44	-
Female	21	83	-	0.44	-	11 (8.5)	107 (91.5)	-	2.27	-
Time of antivenom administration (Hours)										
<1	8 (18)	36 (82)	-	0.15	-	-	22 (100)	-	0.168	-
2–6	33 (22)	117 (78)	3.8 ± 1.4	0.83	0.328	9 (12)	66 (88)	3.84 ± 1.4	0.962	0.35
7–12	13 (21)	49 (79)	9.6 ± 2.1	0.23	0.367	0 (0)	31 (100)	-	0.254	-
>12	23 (31)	51 (69)	53.9 ± 72.7	0.29	0.001 *	4 (16)	21 (84)	53.9 ± 72.7	0.195	0.109
Days of Hospital Stay										
<2	11 (11)	85 (89)	1.5 ± 0.5	0.39	0.75	1 (2)	47 (98)	1.5 ± 0.5	0.39	0.317
3–7	29 (15)	159 (85)	4.4 ± 1.3	1.24	0.0003 *	3 (3)	91 (97)	4.4 ± 1.3	1.24	0.112
>8	37 (66)	19 (34)	18.2 ± 17	0.24	0.19	9 (32)	19 (68)	18.1 ± 17.3	0.2	0.002 *

* Significant difference *p*-value < 0.005.

**Table 5:** Multinomial logistic regression model for predictors of severity in snakebite envenomation in children.

Parameter Estimations								
Severity		B	Error típ.	Wald	*p*-Value	Exp(B)	95% Confidence Interval for Exp(B)	
							Lower Limit	Upper Limit
Severe	Intercept	−5.129	1.779	8.313	0.004			
	Age	0.002	0.103	-	0.985	1.002	0.819	1.226
	Female sex	−0.465	0.995	0.219	0.04	0.628	0.089	4.415
	Male sex	-	-	-	-	-	-	-
	Hospital Stay Days	2.753	0.626	19.353	-	15.689	4.602	53.486
	Non-Medical Practices = No	−1.899	1.128	2.833	0.042	0.15	0.016	1.367
	Non-Medical Practices = Yes	-	-	-	-	-	-	-
Moderate	Intercept	−3.604	1.631	4.881	0.027			
	Age	0.043	0.089	0.235	0.628	1.044	0.877	1.242
	Female sex	0.426	0.83	0.263	0.608	1.531	0.301	7.781
	Male sex	-	-	-	-	-	-	-
	Hospital Stay Days	2.418	0.62	15.188	-	11.225	3.327	37.872
	Non-Medical Practices = No	−0.549	1.027	0.285	0.593	0.578	0.077	4.326
	Non-Medical Practices = Yes	-	-	-	-	-	-	-
Mild	Intercept	−1.884	1.549	1.479	0.224			
	Age	−0.002	0.083	-	0.983	0.998	0.848	1.175
	Female sex	0.133	0.78	0.029	0.865	1.142	0.247	5.273
	Male sex	-	-	-	-	-	-	-
	Hospital Stay Days	1.608	0.605	7.057	0.008	4.992	1.524	16.348
	Non-Medical Practices = No	0.438	1.019	0.185	0.667	1.55	0.211	11.412
	Non-Medical Practices = Yes	-	-	-	-	-	-	-

B: Model Parameter, Exp(B): exponential of the parameter.

## Data Availability

The data presented in this study are available upon reasonable request from the corresponding author.
